# *Metarhizium anisopliae* blastospores are highly virulent to adult *Aedes aegypti*, an important arbovirus vector

**DOI:** 10.1186/s13071-021-05055-z

**Published:** 2021-10-28

**Authors:** Adriano Rodrigues de Paula, Leila Eid Imad Silva, Anderson Ribeiro, Gerson Adriano da Silva, Carlos Peres Silva, Tariq M. Butt, Richard Ian Samuels

**Affiliations:** 1 Laboratório de Entomologia e Fitopatologia, Universidade Estadual do Norte Fluminense Darcy Ribeiro, Campos dos Goytacazes, Rio de Janeiro, 28013-602 Brazil; 2 Departamento de Bioquímica, Universidade Federal de Santa Catarina, Florianópolis, Santa Catarina 88040-900 Brazil; 3 Department of Biosciences, Swansea University, Wales, SA2 8PP UK

**Keywords:** Arbovirus, Dengue, Biological control, Fungus, Pathogen, Blastospores, Conidia

## Abstract

**Background:**

The use of entomopathogenic fungi (EPF) for the control of adult mosquitoes is a promising alternative to synthetic insecticides. Previous studies have only evaluated conidiospores against adult mosquitoes. However, blastospores, which are highly virulent against mosquito larvae and pupae, could also be effective against adults.

**Methods:**

*Metarhizium anisopliae* (ESALQ 818 and LEF 2000) blastospores and conidia were first tested against adult *Aedes aegypti* by spraying insects with spore suspensions. Blastospores were then tested using an indirect contact bioassay, exposing mosquitoes to fungus-impregnated cloths. Virulence when using blastospores suspended in 20% sunflower oil was also investigated.

**Results:**

Female mosquitoes sprayed with blastospores or conidia at a concentration of 10^8^ propagules ml^−1^ were highly susceptible to both types of spores, resulting in 100% mortality within 7 days. However, significant differences in virulence of the isolates and propagules became apparent at 10^7^ spores ml^−1^, with ESALQ 818 blastospores being more virulent than LEF 2000 blastospores. ESALQ 818 blastospores were highly virulent when mosquitoes were exposed to black cotton cloths impregnated with blastospores shortly after preparing the suspensions, but virulence declined rapidly 12 h post-application. The addition of vegetable oil to blastospores helped maintain virulence for up to 48 h.

**Conclusion:**

The results showed that blastospores were more virulent to adult female *Ae. aegypti* than conidia when sprayed onto the insects or applied to black cloths. Vegetable oil helped maintain blastospore virulence. The results show that blastospores have potential for use in integrated vector management, although new formulations and drying techniques need to be investigated.

**Graphical abstract:**

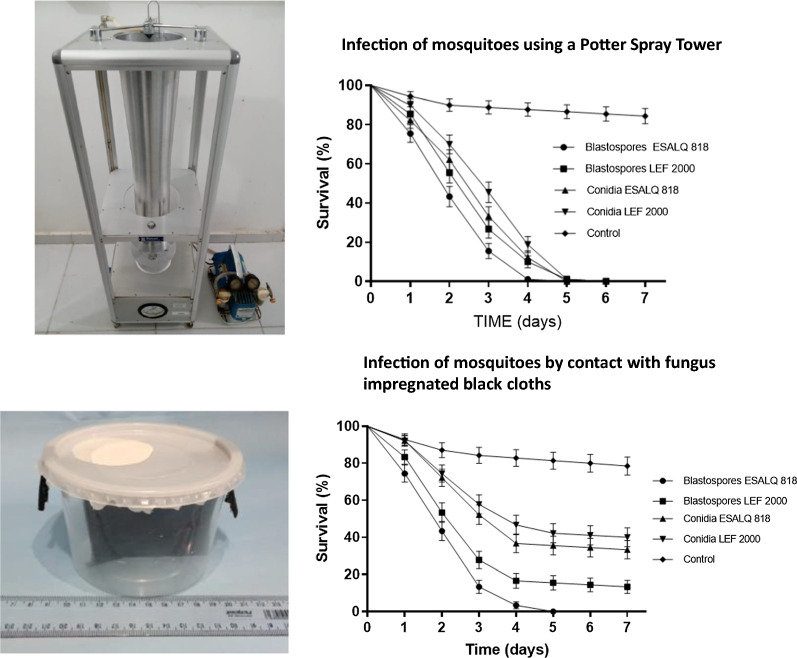

**Supplementary Information:**

The online version contains supplementary material available at 10.1186/s13071-021-05055-z.

## Background

*Aedes aegypti* (Diptera: Culicidae) is a vector of dengue, chikungunya, Zika, and urban yellow fever [1]. In the case of dengue, it has been estimated that 3.9 billion people are at risk of infection in 129 countries [2, 3]. Modeling of dengue virus infections in 2013 gave a figure of 390 million infections per year worldwide (95% credible interval 284–528 million), of which 96 million (67–136 million) cases were manifested clinically [4].

Application of synthetic insecticides is currently the principal method used to control these insects and thus reduce the incidence of diseases [5]. However, the negative effects of using chemical insecticides, such as the selection for resistant genotypes, environmental pollution, and human health concerns, have encouraged the search for alternative methods of mosquito control [6]. One alternative is the use of biological control agents, for example, entomopathogens. Products based on the bacteria *Bacillus thuringiensis israelensis* (Bti) are currently commercialized for the control of larval stages of the mosquito life cycle [7]; however, there are no purely biological control agents available for use against adults. Studies indicate that entomopathogenic fungi (EPF) are promising candidates for the control of adult *Aedes*, *Culex*, and *Anopheles* [8]. However, many studies which propose the use of EPF as a part of integrated vector management (IVM) still need to be evaluated in the field, which includes operational feasibility and economic assessments, to provide evidence to support implementation [9].

The vast majority of EPF sold commercially (targeting agricultural pests) belong to the genera *Metarhizium* and *Beauveria* [10]. The infective propagules consist of either conidia or blastospores, which are produced on solid or liquid substrates, respectively [11]. Blastospores have several advantages over conidia. Firstly, they can be produced quickly, taking 2–3 days, whereas conidial production can take 14–21 days. Some studies have shown conidia to be more virulent than blastospores, whilst others have shown the converse or that they are equally virulent [12–15]. Blastospores have been shown to be more virulent to adult stages than to juveniles, the opposite being true for conidia [16]. When comparing mosquito larvae and pupae, blastospores were more virulent that conidia, independent of mosquito species tested [15, 17, 18].

However, one of the drawbacks with using blastospores for biological control is that they are more susceptible to environmental stress, possibly due to the thinner cell walls, and consequently they are less stable than aerial conidia [11]. All previous studies of EPF targeting adult mosquitoes have focused on evaluating aqueous or oil-formulated conidia, revealing differences in the efficacy of the formulations and virulence of different isolates of *Beauveria* or *Metarhizium* against, for example, *Ae. aegypti* [19], *Aedes albopictus*, *Culex pipiens* [20], *Anopheles gambiae* sensu stricto,* Anopheles arabiensis*, and *Culex quinquefasciatus* [21].

The potential for using black cloths impregnated with *Metarhizium anisopliae* conidia for the control of adult *Ae. aegypti* has been evaluated under laboratory and simulated field conditions by our group [22–24]. Mosquitoes are attracted to land on the fungus-impregnated black cloths, thus becoming infected, and their survival rates are rapidly reduced [25]. Formulating conidia in vegetable oil + isoparaffin significantly increased persistence in the field [24].

Due to the lack of any information on the pathogenicity and virulence of *M. anisopliae* blastospores against adult mosquitoes, the present study was carried out to firstly ascertain whether blastospores were pathogenic to *Ae. aegypti* adults, and then to compare the virulence of the blastospores and conidia of two different *M. anisopliae* isolates. New biological control agents and novel innovative strategies are urgently needed to reduce populations of disease-transmitting mosquitoes.

## Methods

### Mosquito collection and rearing

Adult *Ae. aegypti* used in this study were reared from eggs collected in the field (university campus), as these were considered fitter and more representative of natural populations than the more homogeneous, laboratory-reared mosquitoes. The eggs were collected using ovitraps, which consist of black plastic plant pots (12 cm in diameter × 15 cm in height) with four wooden strips or paddles (3 × 12 cm) placed vertically within the pots, providing highly conducive landing platforms for gravid, ovipositing females (see Additional file 1). Approximately 300 ml of tap water was added to each ovitrap before placing it outdoors at sites protected from rain and direct sunlight close to the university insectary (latitude: −21°45′8.17″ S; longitude: −41°19′49.58″ W).

After 5 days, the paddles with eggs were collected and dried at room temperature for 24 h. To initiate egg hatching, the paddles were submerged in water, and the emergent larvae were maintained in plastic trays (approximately 100 larvae per 100 ml) and fed on freshly ground, autoclaved commercial fish food (Nuvilab, São Paulo, Brazil; 0.05 g/l). Pupae were separated into water-filled beakers and transferred to cages prior to adult emergence. Adults were maintained in cages with 10% sucrose wick feeders. Recently emerged (2–3 days old) females that had been maintained in cages with males were initially anesthetized using a stream of CO_2_ and then placed on top of a glass plate at a temperature of approximately 6 °C with ice packs underneath the plate to maintain the mosquitoes in a dormant state for a maximum of 5 min. The females were then separated from males with the aid of an LED illuminated magnifying glass.

### Fungal isolates

*Metarhizium anisopliae* (isolate ESALQ 818) was obtained from the Universidade de São Paulo—Escola Superior de Agricultura “Luiz de Queiroz” (ESALQ) in Piracicaba, São Paulo, Brazil. Conidia of this isolate have been previously shown to be highly virulent to adult *Ae. aegypti* [19]. *Metarhizium anisopliae* isolate LEF 2000 was obtained from a soil sample in Campos dos Goytacazes, Brazil (latitude: −21°45′8.17″ S; longitude: −41°19′49.58″ W).

### Preparation of conidia

All of the following methods were carried out using a sterile flow hood with materials that had been previously autoclaved (20 min at 121 °C) where appropriate. Both isolates of *M. anisopliae* were initially cultured on Sabouraud dextrose agar (SDA: dextrose 10 g, peptone 2.5 g, yeast extract 2.5 g, agar 20 g in 1 l H_2_O) for 15 days at 27 °C. SDA was autoclaved for 20 min at 121 °C before use. Conidia were then harvested from the solid media using a spatula and suspended in 5 ml of 0.01% aqueous Tween 20. This suspension was used to inoculate 25 g of sterile parboiled rice (autoclaved as above) + 10 ml distilled water in a 250 ml conical flask. The flasks were incubated at 27 °C for 15 days before transferring the inoculated rice to brown paper bags (18 × 9 cm; Fasapel Ltd., São Paulo, Brazil), and humidity was reduced using a forced-air incubator at 34 °C for 24 h before harvesting the conidia using an MR-5 Mycoharvester^®^ (Mycoharvester, UK). Dry conidia were suspended in 0.01% aqueous Tween 20, and the concentration was determined using a Neubauer hemocytometer. On average, 0.1 g of dry conidia was equivalent to approximately 5 × 10^8^ conidia ml^−1^. The fungal concentrations used in assays were 1 × 10^7^ conidia ml^−1^ or 1 × 10^8^ conidia ml^−1^. Viability tests were carried out by plate counting, and only batches with > 90% germination were used in experiments.

### Production of blastospores

Conidia harvested from 15-day cultures were used to initiate the production of blastospores. Blastospores were produced in corn steep liquid medium consisting of 3% (v/v) corn steep liquor (Sigma-Aldrich, Brazil), 4% yeast extract (w/v), and 4% glucose (w/v). Briefly, 500 µl of a conidial suspension (1 × 10^7^ ml^−1^) was added to a 250 ml Erlenmeyer flask containing 50 ml of culture medium. The flask was incubated at 27 °C in an orbital shaker at 152 rpm, and blastospores were harvested after 3 days. The blastospores were separated from the hyphal fragments using a Miracloth filter (Sigma-Aldrich, Brazil), and yield was estimated using a Neubauer hemocytometer. A final concentration of 1 × 10^7^ or 1 × 10^8^ blastospores ml^−1^ was used in the assays for both isolates. For the experiments to test the effects of vegetable oil, blastospores were suspended in 0.01% Tween 20 with or without 20% sunflower oil (Sadia^®^, Brazil). Before use, blastospores suspended in oil + Tween 20 were vigorously agitated using a vortex mixer to form an emulsion, which was immediately applied to the cloths.

### Spraying mosquitoes with blastospore and conidial suspensions

Female *Ae. aegypti* were exposed to *M. anisopliae* (ESALQ 818 or LEF 2000) by spraying with either conidial or blastospore suspensions. Cohorts consisting of 10 mosquitoes were first anesthetized using a stream of CO_2_ for 30 s according to Paula et al. [19]. The anesthetized mosquitoes were then placed on a chilled platform as stated above. Females were then quickly transferred to a Petri dish lined with a sterile filter paper before being sprayed using a Potter tower (Burkart Ltd., UK) with 1 ml of either *M. anisopliae* conidia or blastospores at two different concentrations (1 × 10^7^ or 1 × 10^8^ propagules ml^−1^) in 0.01% (v/v) aqueous Tween 20. Control insects were sprayed with 1 ml of 0.01% Tween 20 only. The number of fungal propagules per cm^2^ targeting the mosquitoes on the filter paper was estimated by randomly sampling five 1 cm^2^ pieces of filter paper after each experiment. Each piece of filter paper was placed in an Eppendorf tube with 1 ml of 0.01% Tween 20, which was then agitated vigorously using a vortex mixer for 1 min. The average number of conidia or blastospores per cm^2^ were estimated using a hemocytometer.

Mosquitoes were then carefully transferred to plastic pots (12 × 7 cm) with mesh netting screen lids (see Graphical abstract). The pots were kept in an incubator (25 °C; 70 ± 10% relative humidity [RH]; 12 h/12 h light/dark). The mosquitoes were fed daily with 10% sucrose offered on filter paper discs placed on the surface of the netting. Mosquito survival was assessed daily for 7 days. These experiments were performed three times for each isolate, with 30 insects used per treatment/isolate (*n* = 90 insects per treatment/isolate; treatment *N* = 180; control *N* = 180).

### Exposing mosquitoes to blastospore-impregnated black cloths at different times after application to the cloths

In this test, ESALQ 818 blastospores were used to impregnate black cloths, to which adult female *Ae. aegypti* were subsequently exposed. Only ESALQ 818 blastospores were used in these experiments, as these propagules were significantly more virulent than LEF 2000 blastospores. The survival of mosquitoes exposed to the cloths at different times after application of blastospores to the cloths was evaluated to monitor the virulence of the inoculum over time.

Black cotton cloths (8 × 6 cm) were autoclaved at 121 °C for 15 min and then impregnated with blastospores by brushing each side of the cloth with suspensions of 5 ml of 1 × 10^7^ blastospores ml^−1^ in 0.01% aqueous Tween 20. One set of cloths was immediately used for bioassays, whilst two other sets of cloths were left to dry at room temperature (maximum: 26.9 °C minimum: 23.8 °C; maximum: 85% RH and minimum: 71.6% RH) for either 12 h or 24 h. Three sets of control cloths were treated with 5 ml of 0.01% aqueous Tween 20. One set was used immediately and the other two sets were left to dry for either 12 h or 24 h.

The bioassay was carried out by suspending a single black cloth vertically inside each plastic pot (12 × 7 cm) before releasing 10 female mosquitoes into the pot. This methodology was the same as that developed by Paula et al. [25]. Female mosquitoes were previously anesthetized as described above before placing in the pots. The mosquitoes were able to move freely around the pot, which was kept in an incubator (25 °C; 70 ± 10% RH; 12 h/12 h light/dark). The insects were fed daily with 10% sucrose offered on filter paper discs placed on the surface of the white mesh netting of the lids. After a period of 48 h, the black cloths were carefully removed from the plastic pots. Mosquito survival was assessed daily for 7 days.

For each time point (0 h, 12 h, and 24 h after impregnating the cloths with fungus), 30 mosquitoes were used (10 per pot). Mosquitoes were also exposed to control cloths at the three different time points. Therefore, a total of 90 mosquitoes were used per experimental treatment (three time periods) and 90 controls (three time periods). The experiments were carried out three times (*N* = 270 blastospore-treated mosquitoes; *N* = 270 controls).

### Application of blastospore suspensions in oil to black cloths

This bioassay was carried out as stated above, except that the blastospores were suspended in 20% sunflower oil + aqueous Tween 20 (0.01%) and then applied to black cloths as an emulsion. The survival rates of mosquitoes exposed to these cloths were then compared to mosquitoes exposed to blastospores suspended in Tween 20 only. This experiment evaluated the virulence of the blastospores applied to the cloths at different times following application to the cloths. Mosquitoes were placed in pots with blastospore-impregnated cloths at 0 h (immediately after preparing the cloths), 12 h, 24 h, and 48 h after applying blastospores to the cloths. Control applications of 20% sunflower oil + Tween 20 without blastospores or Tween 20 without blastospores were also used at each time point. Three cohorts (pots with 10 mosquitoes each) were used for each time point. Therefore, a total of 120 mosquitoes were used per experimental treatment (four time periods) and 120 controls (four time periods). The experiments were carried out three times (*N* = 360 blastospore-treated mosquitoes; *N* = 360 controls).

### Blastospore germination and colony growth

Freshly harvested ESALQ 818 and LEF 2000 blastospores were suspended in 0.01% aqueous Tween 20 with and without 20% sunflower oil, vortex mixed vigorously for 1 min, and then 100 μl aliquots of the suspensions were inoculated onto SDA plates. The plates were then incubated for 6 h at 27 °C. The germination rates after this time period were evaluated using an inverted microscope (Biovera, São Paulo, Brazil). Three fields of view were selected randomly for each plate counted and evaluations were carried out three times to calculate the mean germination rates. A 6-h incubation time was chosen for counting as germination rates were normally higher than 50% but less than 100% in preliminary experiments. This time period allowed comparisons to be made between the different treatments and isolates.

Fungal radial growth experiments were performed using both ESALQ 818 and LEF 2000 blastospores at a concentration of 1 × 10^6^ blastospores ml^−1^ with and without 20% sunflower oil in 0.01% aqueous Tween 20, prepared as stated above. For this experiment, a 15 µl aliquot of each suspension was inoculated in the center of each Petri dish (9 cm diameter) containing SDA. The plates were incubated at 27 °C and 70 ± 10% RH for a total of 10 days. Three plates were used for each treatment and all experiments were performed three times. Radial growth (mm) was estimated daily using Vernier calipers, measuring growth rates from the center of the colony to the external border.

### Statistical analysis

All data analyses were performed with GraphPad Prism 6.0 software package (GraphPad Software, San Diego, CA, USA). For survival data, the homogeneity of the repetitions was analyzed using the log-rank test at a 95% significance level. Homogenous results were then pooled for survival curve analysis. The average survival time (*S*_50_) was calculated using the Kaplan–Meier method [33]. Statistical differences between the survival curves of different treatments were compared using the log-rank test. The results for all the control groups were pooled and only one survival curve was shown for each figure.

Comparison of *Ae. aegypti* end-point survival percentages for the different treatments was assessed using one-way analysis of variance (ANOVA). Mean germination rates and mean radial growth were compared using unidirectional analysis of variance. Differences between groups were considered significant if the *P* value was ≤ 0.05 (Duncan’s post hoc test).

## Results

**Fig. 1:**
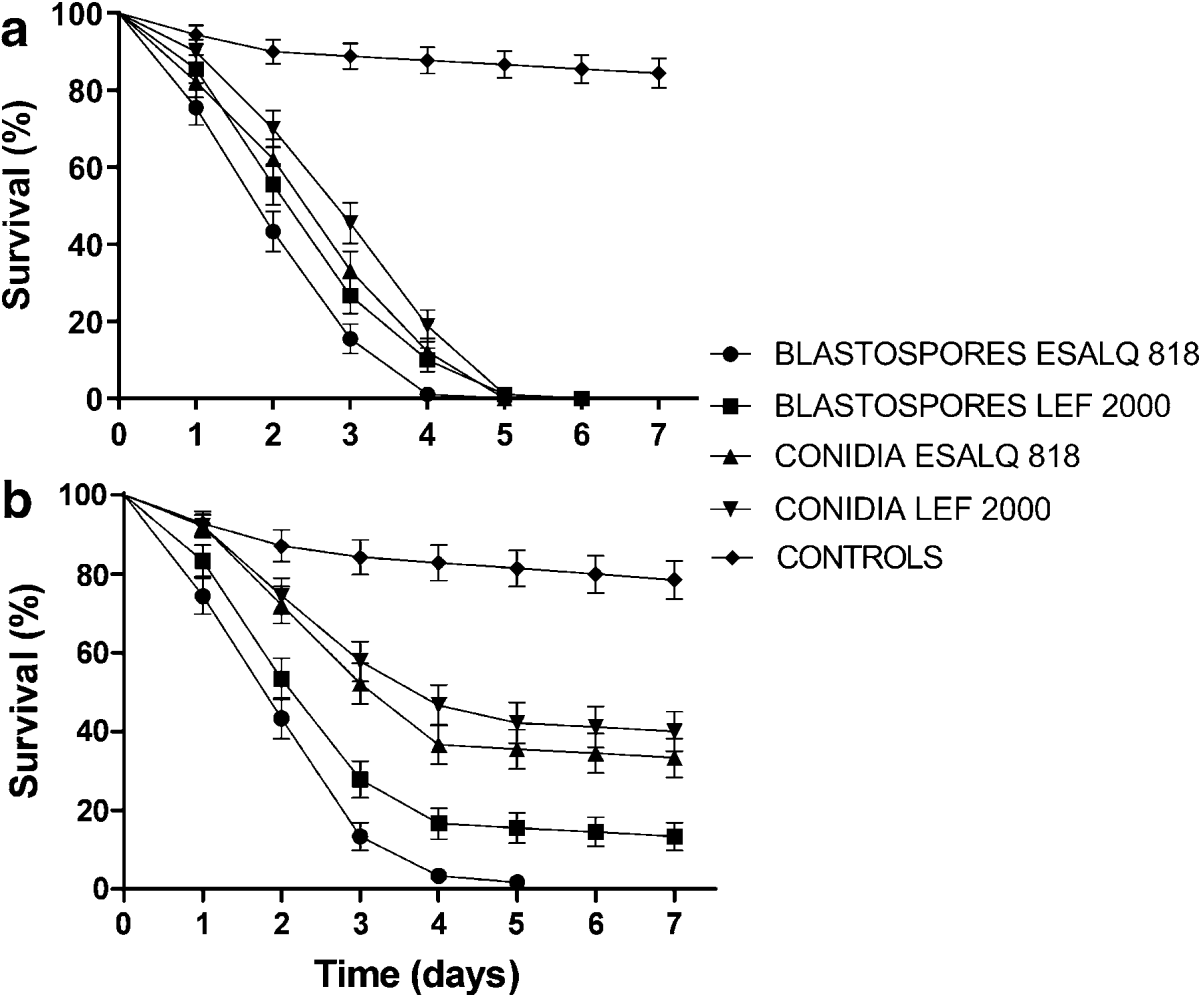
**a** Survival curves of *Aedes aegypti* females sprayed with conidia or blastospore suspensions of two *Metarhizium anisopliae* isolates (ESALQ 818 and LEF 2000) at a concentration of 1 × 10^8^ propagules ml^−1^. **b** Survival curves of *Ae. aegypti* females sprayed with conidia or blastospore suspensions of two *Metarhizium anisopliae* isolates (ESALQ 818 and LEF 2000) at a concentration of 1 × 10 propagules ml^−1^. Control data for all treatment groups were combined and are represented as a single survival curve. Error bars are ± SD (standard deviation of the mean)

The first bioassay employed a spray application technique to evaluate the virulence of blastospores and conidia of both isolates. The results demonstrated that both isolates and both types of propagules were highly virulent when mosquitoes were sprayed with 1 × 10^8^ propagules ml^−1^ (Fig. 1a). This concentration of propagules was estimated to give a coverage of 1.7 × 10^6^ ± 1.4 × 10^5^ conidia/blastospores cm^2^ when counting propagules from the filter paper onto which the mosquitos were placed on the platform of the Potter tower.

The lowest median survival time (*S*_50_) was seen when mosquitos were sprayed with ESALQ 818 blastospores (2 days), whilst a value *S*_50_ of 3 days was seen for the three other treatments: ESALQ 818 conidia, LEF 2000 blastospores and LEF 2000 conidia (Table 1). Evaluations of survival rates when using this concentration of propagules was performed on day 3, as all fungus-treated mosquitoes had died by day 7. When comparing survival rates for all treatments and controls on day 3 of the experiment, ESALQ 818 blastospores were seen to be significantly more virulent (*F*_(4, 14)_ = 75.15, *P* < 0.0001) with only 15% of the insects surviving at this time (Table 1). The survival rates of mosquitoes sprayed with LEF 2000 blastospores or ESALQ 818 conidia was 26.6% and 33.3%, respectively, with no significant difference between these two treatments. LEF 2000 conidia were the least virulent, with 45% survival recorded on day 3.

**Table 1:** *Aedes aegypti* survival rates on day 3 and day 7 following different treatments

Treatments/isolate	Day 3		Day 7	
	Survival (%) ± SD 1 × 10^8^ conidia^−1^	*S*_50_ (days)	Survival (%) ± SD 1 × 10^7^ conidia^−1^	*S*_50_ (days)
Blastospores ESALQ 818	15.5 ± 3.51a	2	0	2
Conidia ESALQ 818	33.3 ± 5.29b	3	36.6 ± 8.45 b	4
Blastospores LEF 2000	26.6 ± 7.81b	3	16.6 ± 9.39 a	3
Conidia LEF 2000	45.5 ± 6.65c	3	42.2 ± 6.54 c	4
Controls	88 ± 1.52d	nd	87.7 ± 2.14 d	ND

Results are shown as the mean survival rates (% ± SD) after spraying mosquitoes with 1 × 10^8^ ml^−1^ (conidia or blastospores) of two isolates (ESALQ 818 and LEF 2000) on day 3 or day 7 following spraying of mosquitoes with 1 × 10^7^ ml^−1^ (conidia or blastospores)

The mean survival percentages followed by different letters indicate statistical differences when comparing values (columns) using ANOVA followed by Duncan’s post hoc (5% level). Control data for each treatment group were combined, and a single mean survival rate was calculated

*nd* not determined

In order to more clearly differentiate the virulence of the isolates and propagules, spray applications were carried out at a lower concentration (1 × 10^7^ propagules ml^−1^). This concentration of propagules was estimated to give a coverage of 8 × 10^5^ ± 1.3 × 10^5^ conidia/blastospores cm^2^ when counting propagules from the filter paper onto which the mosquitos were placed on the platform of the Potter tower.

Bioassays at this concentration of propagules demonstrated that ESALQ 818 blastospores were significantly more virulent than ESALQ 818 conidia, LEF 2000 blastospores, or LEF 2000 conidia (*X*^2^ = 138.7, *P* < 0.0001). ESALQ 818 blastospore applications resulted in 100% mortality on day 5 of the bioassay (Fig. 1b). In these experiments, the survival rates for the controls were on average 87%. The mean end-point survival rates (on day 7) were significantly different when comparing all treatments, including the controls (Table 1). The ANOVA also showed that mean survival rates for all fungal treatments and their respective controls were significantly different (*F*_(4, 14)_ = 465.3, *P* < 0.0001). The lowest *S*_50_ value was seen for ESALQ 818 blastospores (2 days). The *S*_50_ for LEF 2000 blastospores was 3 days, whilst the other two fungal treatments, LEF 2000 conidia and ESALQ 818 conidia, both had an *S*_50_ value of 4 days (Table 1). This information was important for the development of the next bioassay testing the virulence of blastospores applied to black cloths to which the mosquitoes were subsequently exposed. As the aim of the investigation was to evaluate blastospores as putative biological control agents for use against adult mosquitoes, only ESALQ 818 blastospores (most virulent propagules) were used in the following bioassays.

**Fig. 2:**
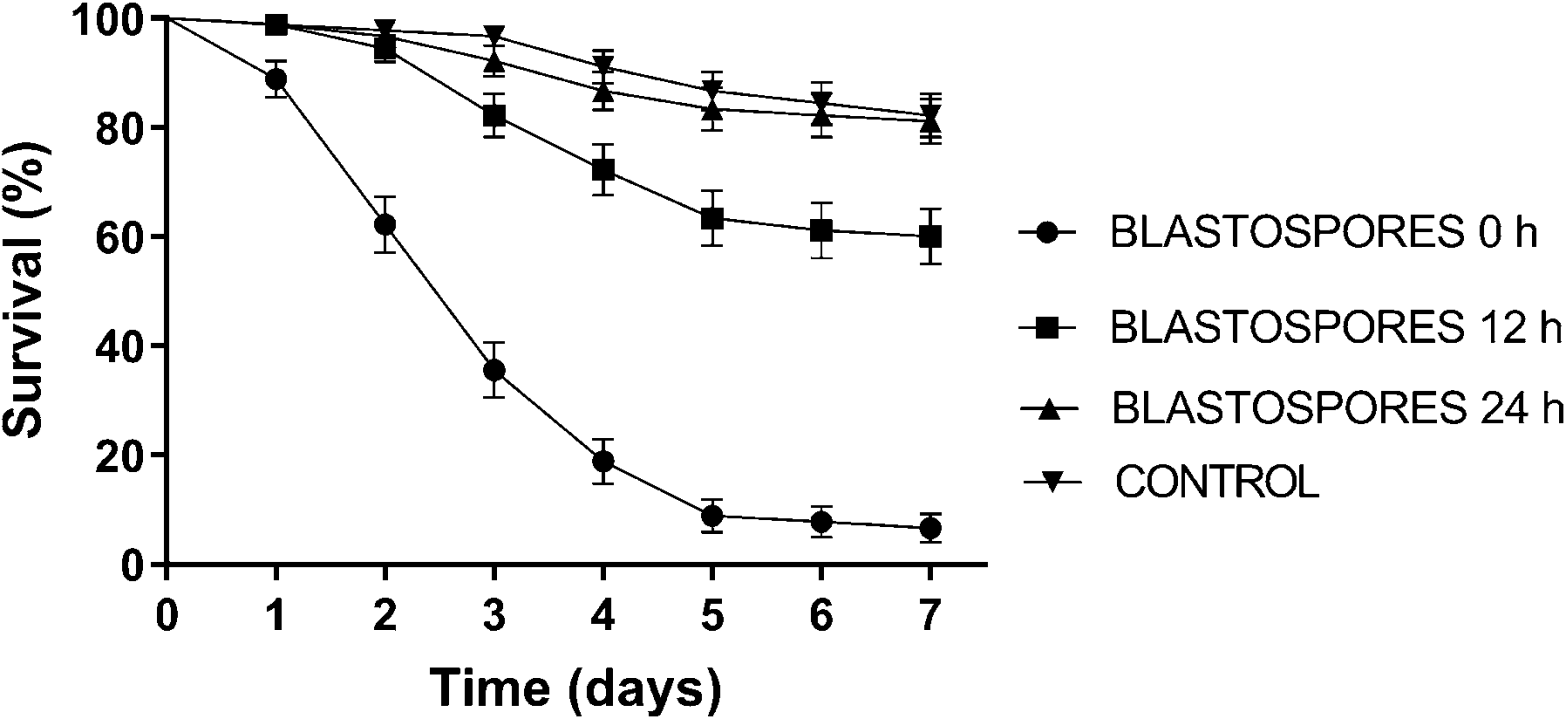
Survival curves of *Aedes aegypti* females following exposure to ESALQ 818 blastospore (1 × 10^7^ ml^−1^)-impregnated black cotton cloths immediately after applying the blastospore suspensions to the cloths (0 h), 12 h after preparing the cloths, and 24 h after preparing the cloths. Control data for all treatment groups were combined and represented as a single survival curve. Error bars are ± SD (standard deviation of the mean)

Black cotton cloths were impregnated with 1 × 10 blastospores ml^−1^ in Tween 20 (0.01% v/v). This concentration of propagules was estimated to give a coverage of 9 × 10^6^ ± 8 × 10^5^ blastospores cm^2^ when applying 5 ml of the fungal suspension using a paintbrush to the cloths. When exposing mosquitoes to blastospore-impregnated black cloths, a significant reduction in survival rates (*X*^2^ = 207.5, *P* < 0.0001) was observed only when mosquitoes were placed in pots with cloths that had been recently treated with blastospores (0 h). The survival rate for this treatment was 8.8%, whereas mosquito survival following exposure to cloths 12 and 24 h after application of blastospores resulted in 61% and 81% survival, respectively (Fig. 2). Only the 0-h treatment generated a *S*_50_ value, which was 3 days (Table 2). The endpoint survival rates for all treatments were significantly different (*F*_(3, 11)_ = 956.3, *P* < 0.0001); however, the survival rates of mosquitoes exposed to cloths 24 h after application of the fungus were not significantly different from the control survival rates.

**Table 2:** *Aedes aegypti* survival rates following exposure to blastospores at different times after applying the suspensions to the cloths

Drying time	Blastospores 0 h	Blastospores 12 h	Blastospores 24 h	Control
Survival (%) ± SD	8.8 ± 9.97a	61.1 ± 4.32b	81.1 ± 1.61c	82.2 ± 2.36 c
*S*_50_	3	nd	nd	nd

End-point survival rates (% ± SD) and median survival times (*S*_50_) of female *Ae. aegypti* 7 days after exposure to cloths impregnated with ESALQ 818 blastospores at a concentration of 1 × 10^7^ propagules ml^−1^. The cloths were placed in the pots immediately (0 h), 12 h, or 24 h after applying blastospores to the cloths. Mean survival percentages followed by different letters indicate statistical differences when comparing values using ANOVA followed by Duncan’s post hoc (5% level). Control data for all treatment groups were combined, and a single mean survival rate was calculated

*nd* not determined

**Fig. 3:**
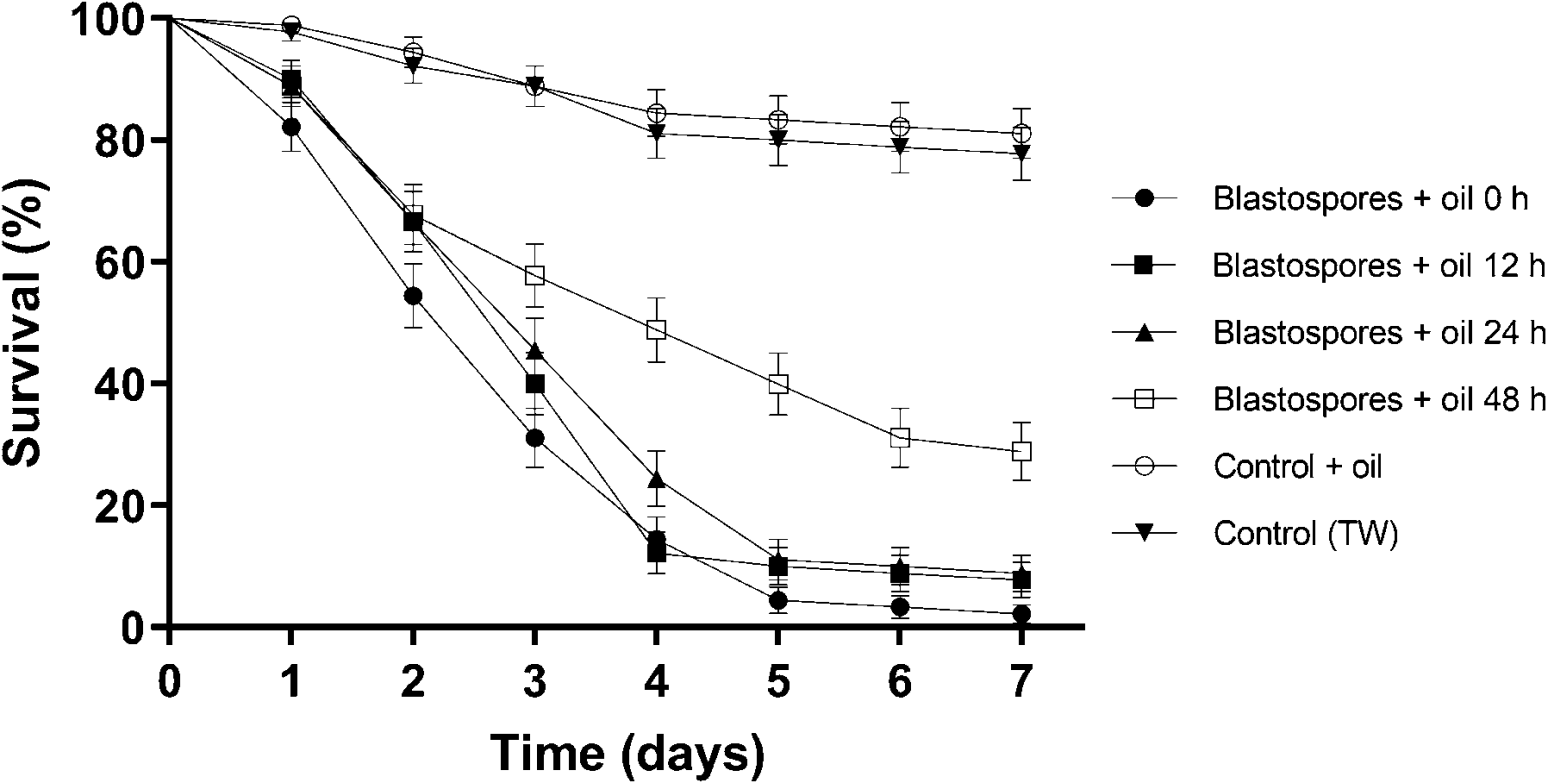
Survival curves of *Aedes aegypti* females following exposure to ESALQ 818 blastospore (1 × 10^7^ ml^−1^)-impregnated black cotton cloths with and without the addition of 20% sunflower oil to the suspensions. Mosquitoes were exposed to the cloths immediately after application of fungi to the cloths (0 h), or 12 h later, 24 h later, or 48 h later. Control data for all treatment groups were combined and represented as a single survival curve. Error bars are ± SD (standard deviation of the mean)

In an attempt to improve blastospore efficiency, these propagules were then tested following the addition of 20% vegetable oil to the suspensions. This oil + Tween 20 formulation was tested at four time periods post-application to the cloths: 0, 12, 24, and 48 h. Mosquito survival curves are shown in Fig. 3. The sharpest decline in survival rates was seen when exposing mosquitoes to blastospore-impregnated cloths immediately after applying the suspension to the cloths, as had been previously observed for blastospores in Tween 20 only (Fig. 2). For mosquitoes exposed to cloths immediately after the application of blastospores (0 h), survival declined rapidly, with only 4% of the mosquitoes remaining alive by day 7 of the experiment (Fig. 3); this value was significantly lower than all other treatments when comparing survival curves using log-rank analysis (*X*^2^ = 316.6, *P* < 0.0001). Exposing mosquitoes to cloths 12 h and 24 h after application of blastospores resulted in similar survival curves, with 8% and 11% end-point survival rates, which were not significantly different from each other (Table 3). Mosquitoes exposed to blastospores 48 h after preparation of the cloths, resulted in a survival rate of 28% on the seventh day of evaluation (Fig. 3 and Table 3). Although this survival rate was significantly higher than that of the other time points, it was still significantly lower than that of the controls (Table 3: *F*_(20, 6)_ = 700.1, *P* < 0.0001). Although the end-point survival rates were significantly lower for the 0-h treatment group, *S*_50_ values for 0 h, 12 h, and 24 h were all 3 days. The *S*_50_ for blastospores + oil tested 48 h after applying the formulation to the cloths was 4 days (Table 3).

**Table 3:** *Aedes aegypti* survival rates following exposure to blastospores formulated in oil at different times after preparation

Treatments	Mean survival (%) ± SD	*S*_50_ (days)
Blastospores + oil 0 h	4 ± 9.75a	3
Blastospores + oil 12 h	8.8 ± 10.6b	3
Blastospores + oil 24 h	11.1 ± 8.37b	3
Blastospores + oil 48 h	28.8 ± 7.9c	4
Control + oil	81.1 ± 2.76d	nd
Control (TW)	82.2 ± 2.28d	nd

Survival rates (% ± SD) and median survival times (*S*_50_) of female *Ae. aegypti* 7 days after exposure to cloths impregnated with ESALQ 818 blastospores at a concentration of 1 × 10^7^ propagules ml^−1^, with and without the addition of 20% sunflower oil to the suspensions. Mosquitoes were exposed to the cloths immediately after application of fungi to the cloths (0 h) or 12 h, 24 h, or 48 h later. Mean survival percentages followed by different letters indicate statistical differences when comparing values using ANOVA followed by Duncan’s post hoc (5% level). Control data for each oil treatment group (four data sets) were combined, and the single mean survival rate was calculated

*nd* not determined, *TW* Tween 20

The germination and growth rates of blastospores of both isolates were similar when suspended in Tween 20 only or Tween 20 + sunflower oil (see Additional file 1: Table S1 and Figure S1).

## Discussion

This study shows for the first time that *M. anisopliae* blastospores are pathogenic to adult *Ae. aegypti* and that blastospores of both fungal isolates tested here were more virulent than conidiospores. The initial tests were carried out by spraying mosquitoes with conidial and blastospore suspensions, in order to confirm and compare the virulence of the fungal propagules. Our research group has used this spray infection technique for certain types of bioassays, but the main aim of our research is to develop traps which reduce adult mosquito survival by attracting the mosquitos to land on fungus-impregnated surfaces [22, 23]. Mosquitoes are attracted to dark surfaces, and studies have shown that *Ae. aegypti* females land on fungus-impregnated black cloths at the same rate as on untreated cloths [25]. Although we do not discount the option of space spraying of fungi, innovative strategies could prove to be more effective in controlling mosquito adult populations to reduce arbovirus transmission.

In the search for highly virulent isolates or propagule types, it is important to consider that mosquitoes probably land on fungus-impregnated surfaces for short periods, but even short-term contact with the fungus could reduce vectorial capacity. The extrinsic incubation period for dengue transmission by *Ae. aegypti* is between 8 and 12 days [26]. Even though it is not crucial to rapidly kill virus vectoring mosquitoes that have taken their first blood meal, it is still preferable to kill them quickly. Mosquitoes in a range of nutritional states and levels of virus development in their bodies are also targets for EPF. Entomopathogenic fungal infections can reduce vectorial capacity before killing the host mosquito by reducing propensity to feed and fecundity [27].

Previous studies have shown that blastospores are more virulent than conidia when tested against *Ae. aegypti* larvae [15] or pupae [18]. Although it is not clear exactly why blastospores are more virulent than conidia to these aquatic stages of the mosquito life cycle, blastospores are hydrophilic and are thus more easily dispersed in an aquatic environment than hydrophobic conidia, which require the use of surfactants to disperse in water. Another important factor is that blastospores produce large amounts of mucilage, which improves adhesion to the host integument [15]. Blastospores germinate faster than conidia, which can also be an advantage in initiating disease [28]. Rapid germination and penetration of the cuticle enables the fungus to reduce its exposure time to highly damaging UV radiation and low relative humidity [29, 30]. The results of the current study demonstrated that blastospores in water were highly virulent only when used immediately after application to the cloths. However, the addition of sunflower oil to the suspensions resulted in significant improvements in the maintenance of blastospore virulence.

Oil formulations have been previously shown not only to increase conidial shelf life [31] but also to protect conidia in the field from adverse environmental conditions such as low relative humidity [32]. Formulation of conidia in oil was one of the foundations of the success of *Metarhizium* (Green Muscle^®^) for the control of the desert locust (*Schistocerca gregaria*) in Africa [33]. Here, we decided to test blastospores suspended in 20% sunflower oil + Tween 20 applied to the black cloths. However, before using oil-formulated blastospores against mosquitoes, we first checked whether the oil had any adverse effects on blastospore germination or fungal growth. Blastospores germinated at similar rates in the presence or absence of oil, whilst the growth rates of the fungus with and without oil were not significantly different (see Additional file 1: Table S1; Figure S1).

The deployment of black cloths impregnated with conidia has been shown to be a promising strategy for reducing mosquito survival. In one of the first studies to investigate the use of fungus-impregnated black cloths in human habitations, *Beauveria bassiana* was shown to infect and kill significant numbers of *Anopheles stephensi* adults [34]. More recently, Paula et al. [23] tested black cloths impregnated with *M. anisopliae* conidia in rooms simulating a human habitation. Fungus-impregnated cloths deployed in association with an attractive lure (AtrAedes^®^) reduced mosquito survival over a 7-day period to 32%, whilst black cloths impregnated with *M. anisopliae* conidia without lures resulted in a 48% survival rate. This approach is highly promising as an alternative to chemical control methods.

Blastospores are highly virulent against adult *Ae. aegypti*, and the addition of vegetable oil helped maintain virulence for up to 48 h after preparation of fungal suspensions. It is possible that the oil helps maintain the integrity of the blastospores, which are sensitive to desiccation [35] following application to the black cloths. Several hypotheses have been proposed to explain how the addition of oil enhances the efficiency of conidia to target pests. A thin oil layer might prevent the desiccation of fungal propagules by slowing evaporation, thus giving conidia more time to germinate and infect the host [36]. Oils are also known to disrupt the protective layer of epicuticular waxes on the insect cuticle or leaching of cuticle compounds, diluting antifungal compounds present in the epicuticle and thus stimulating germination and subsequent infection [37]. It has also been reported that oil formulations improve adhesion of spores to the lipophilic insect cuticle [38]. Spores may also disseminate more easily to infect susceptible parts of the insect body, such as the intersegmental membranes, when formulated in oil [37, 38]. When blastospores were suspended in 20% vegetable + aqueous surfactant (Tween 20), the emulsion, which was subsequently applied to black cloths, extended the effectiveness of the blastospores over time. It is also interesting to note that fungal virulence can be affected by factors such as culture conditions (nutrients, pH, temperature) [39, 40]. Conidia produced on rice grains were more virulent to *Ae. aegypti* larvae than those produced on standard laboratory media such as Sabouraud dextrose agar [41]. We are currently investigating the effects of different media on blastospore virulence. Following production of the blastospores, it will be necessary to study drying techniques, which are known to improve shelf life [42] .

## Conclusions

Blastospores were more virulent than conidia to adult female *Ae. aegypti* when sprayed onto the insects. Blastospores were also virulent when applied to black cloths onto which mosquitoes landed and became infected. The addition of vegetable oil clearly enhanced the virulence of blastospores, suggesting that it improved adhesion to the insect cuticle and probably created a microclimate conducive for germination. Although these results are preliminary, they show that blastospores have potential for use in integrated vector management. However, new formulations and drying techniques need to be investigated to maintain virulence for longer periods under simulated field conditions.

## Supplementary Information

**Additional file 1: Table S1.** Percentage germination of *Metarhizium anisopliae* (ESALQ 818 and LEF 2000) blastospores when formulated with and without sunflower oil. **Fig S1.** Radial growth (mm) of *Metarhizium anisopliae* (ESALQ 818 and LEF 2000) blastospores formulated with and without sunflower oil (20%) over a 10 day period. Photograph of ovitrap used to collect *Aedes aegypti* eggs in the field.

## Data Availability

All data is available from the corresponding author on request.
