# Supplementary material for: Metarhizium anisopliae blastospores are highly virulent to adult Aedes aegypti, an important arbovirus vector
Source: Parasit Vectors. 2021 Oct 28;14:555. doi: 10.1186/s13071-021-05055-z (PMC8555014; doi:10.1186/s13071-021-05055-z)
Supplement: Supplementary file 1 — Additional file 1: Table S1. Percentage germination of Metarhizium anisopliae (ESALQ 818 and LEF 2000) blastospores when formulated with and without sunflower oil. Fig S1. Radial growth (mm) of Metarhizium anisopliae (ESALQ 818 and LEF 2000) blastospores formulated with and without sunflower oil (20%) over a 10 day period. Photograph of ovitrap used to collect Aedes aegypti eggs in the field. [file 13071_2021_5055_MOESM1_ESM.doc]

**Additional File 1: Paula AR et al.,**

*Metarhizium anisopliae* blastospores are highly virulent to adult *Aedes aegypti*, an important arbovirus vector

**Influence of oil formulation on blastospore germination and development**

Germination rates for ESALQ 818 blastospores suspended in sunflower oil and 0.01% Aq. Tween for 24 h was 83% and 81%, respectively (Table S1). The germination rates of LEF 2000 blastospores suspended in sunflower oil and 0.01% Aq. Tween was 77% and 79%, respectively. There were no significant differences (*P*>0.01) between the treatments (Table S1). Similarly, radial growth of fungal cultures initiated using blastospores suspended with and without sunflower oil was similar for both isolates (Fig S1). Mean radial growth on day 10 showed no significant differences between treatments (F_(3,11)_ = 3.074, *P*> 0.01).

**Table S1** Percentage germination of *Metarhizium anisopliae* (ESALQ 818 and LEF 2000) blastospores when formulated with and without sunflower oil

| **Treatment** | **ESALQ 818** | **Isolado LEF 2000** |
| --- | --- | --- |
| Blastospores + Tween | 81.4 ± 7.09 | 79.8 ± 14.50 |
| Blastospores + Oil | 83.8 ± 26.73 | 77.1 ± 32.47 |

Note: The results are shown as means ± SD. There were no significant differences between any of the treatments.

**Fig S1** Radial growth (mm) of *Metarhizium anisopliae* (ESALQ 818 and LEF 2000) blastospores formulated with and without sunflower oil (20%) over a 10 day period


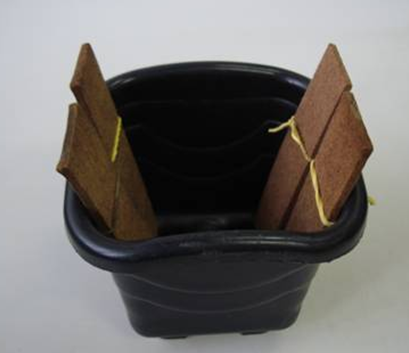


Ovitrap used to collect *Aedes aegypti* eggs in the field.
